# Empirical Bayesian models for analysing molecular serotyping microarrays

**DOI:** 10.1186/1471-2105-12-88

**Published:** 2011-03-31

**Authors:** Richard Newton, Jason Hinds, Lorenz Wernisch

**Affiliations:** 1 MRC Biostatistics Unit, Robinson Way, Cambridge, CB2 0SR, UK; 2 Bacterial Microarray Group, Division of Clinical Sciences, St. George's, University of London, Cranmer Terrace, London, SW17 0RE, UK

## Abstract

**Background:**

Microarrays offer great potential as a platform for molecular diagnostics, testing clinical samples for the presence of numerous biomarkers in highly multiplexed assays. In this study applied to infectious diseases, data from a microarray designed for molecular serotyping of *Streptococcus pneumoniae *was used, identifying the presence of any one of 91 known pneumococcal serotypes from DNA extracts. This microarray incorporated oligonucleotide probes for all known capsular polysaccharide synthesis genes and required a statistical analysis of the microarray intensity data to determine which serotype, or combination of serotypes, were present within a sample based on the combination of genes detected.

**Results:**

We propose an empirical Bayesian model for calculating the probabilities of combinations of serotypes from the microarray data. The model takes into consideration the dependencies between serotypes, induced by genes they have in common, and by homologous genes which, although not identical, are similar to each other in sequence. For serotypes which are very similar in capsular gene composition, extra probes are included on the microarray, providing additional information which is integrated into the Bayesian model. For each serotype combination with high probability, a second model, a Bayesian random effects model is applied to determine the relative abundance of each serotype.

**Conclusions:**

To assess the accuracy of the proposed analysis we applied our methods to experimental data from samples containing individual serotypes and samples containing combinations of serotypes with known levels of abundance. All but two of the known serotypes of *S. pneumoniae *that were tested as individual samples could be uniquely determined by the Bayesian model. The model also enabled the presence of combinations of serotypes within samples to be determined. Serotypes with very low abundance within a combination of serotypes can be detected (down to 2% abundance in this study). As well as detecting the presence of serotype combinations, an approximate measure of the percentage abundance of the serotypes within the combination can be obtained.

## Background

Microarrays are an experimental method for detecting the presence or absence of multiple genes within a sample simultaneously, through specific binding to an array of high-density probes. They therefore have a diagnostic potential in a number of areas including that of infectious diseases. A microarray containing probes for genes specific to different strains of an organism can detect the presence of a particular strain of the organism in a clinical sample according to which of the probes have an elevated signal. Diagnostic testing by microarray is potentially quicker, easier and more reliable than established tests [1-3]. It also has the scope for detecting a range of organisms in a sample in a single test.

A microarray for molecular serotyping of the bacterium *Streptococcus pneumoniae *was designed to detect 91 known serotypes of the pneumococcus from DNA extracts [4]. Clinical samples may contain more than one serotype of *S. pneumoniae*. A key feature of molecular serotyping by microarray, spurring interest in the method, is that rapid detection of multiple serotypes in a clinical sample should be feasible. In addition it should be possible to quantify the relative abundance of each of the serotypes in the sample. Achieving these two goals with established serotyping methods is either prohibitively time consuming or simply not possible. Here we propose a statistical analysis of the *S. pneumoniae *microarray data that achieves both these objectives, identifying both the serotype(s) and their relative abundance in samples.

The *Streptococcus pneumoniae *molecular serotyping microarray was designed with multiple probes representing all the known capsular polysaccharide synthesis genes. In the rest of this paper we refer to these genes as *cps *genes and these genes' probes as the CPS probes. The *cps *genes encode the proteins and enzymes that biosynthesise and assemble the capsular polysaccharide. Serotyping was classically established through cross-reactivity with typing antisera which discriminate each serotype due to structural differences in the capsular polysaccharide. The serotype is relevant because the capsular polysaccharide represents the interface between bacterium and host and so is associated with immunity and invasive disease and forms the basis of polyvalent vaccines currently available. Each serotype of the pneumococcus contains a small subset of the *cps *genes, ranging in number from 1 to 22 [5-7], which determines the nature and structure of the capsule polysaccharide, and thus the serotype may be determined at the genetic level by the combination of *cps *genes present.

For this reason the *cps *genes have been sequenced for all 91 serotypes. Most of the serotypes are not fully sequenced. The design of probes for the array is therefore essentially limited to these genes. However, a number of serotypes may have very similar or even identical combinations of *cps *genes present. Therefore additional probes on the microarray test for more subtle genetic differences between key *cps *genes of such serotypes. In the rest of this paper we refer to these additional probes as STIDs. One problem that the statistical analysis needed to address was the integration of the CPS probe data and the STIDs probe data.

The technique presents several further analysis problems. Fluorescent intensity signals from microarray probes for genes indicate gene abundance only indirectly. Such signals are disturbed by a variety of random factors which are difficult to control: from variation in the DNA extraction to variation in the binding of the DNA to its oligonucleotide probe. Concerning the identification of genes, it is sometimes difficult to design probes which are entirely specific to a particular gene. Due to gene homology and the overall similarity of their DNA sequences, a probe for one gene may bind to the DNA from a different gene (cross-hybridisation). In addition, the genomic DNA of the organisms of interest can be contaminated by DNA from the host and from other commensal or pathogenic organisms.

In the following we develop an empirical Bayesian statistical model for calculating the probabilities of serotype combinations based on the data from the serotyping microarrays. We first set up likelihoods for gene binding depending on microarray log intensities for *cps *genes. Then, likelihoods of serotype combinations depending on gene binding and incorporating cross-hybridisation effects are described. Further, likelihoods for serotypes depending on log intensities of STID probes are provided. Finally, all these likelihoods are put together to give a likelihood of serotype combinations depending on log intensities from CPS probes and STID probes. Combined with a prior on serotype combinations this allows us to infer a posterior probability for serotype combinations, apart from a normalising constant. Some of the hyperparameters of the model are estimated in an empirical Bayes fashion from the microarray data. Since there are exponentially many combinations of serotypes, we use a heuristic to limit the number of combinations to a subset of serotypes and serotype combinations with a potential for high probabilities.

The second objective of the analysis is to quantify the relative abundance of the serotypes in the sample. So in the final part of the methods section we describe a Bayesian random effects model for estimating abundances of serotypes for a fixed combination of serotypes.

In order to assess the accuracy of our experimental and statistical approach, we analysed microarray data from samples of the 91 known serotypes of the bacterium *S. pneumoniae*. We refer to these 91 microarrays as *reference arrays*. In the first assessment of the method the task was to detect the single serotype present in the sample when applied to the 91 reference arrays. Then, in order to assess the capability of the method to detect combinations of more than one serotype, four additional sets of microarray data were produced using a combination of three or five serotypes in known abundance in a sample. The latter microarrays, to which we refer as *spike-in experiments*, also allow us to assess the accuracy of the Bayesian random effects model in predicting abundances of serotypes.

## Methods

The first part of the methods section describes the data; the *Streptococcus pneumoniae *microarray and the datasets used in this article. In the second part we develop a probabilistic model for calculating the likelihood of a combination of serotypes given the binding intensities measured for CPS probes and STID probes on a microarray. In the third part of the methods we describe a Bayesian random effects model for estimating the abundance of each serotype in a combination.

### Data

#### Streptococcus pneumoniae microarray

The B*μ*GS SP-CPSv1.1.0 microarray [4] is a custom designed microarray on the Agilent SurePrint platform [8], printed in the 8 × 15K format and comprised primarily of 60mer oligonucleotides probes. These microarrays were hybridized as two colour arrays but the two channels were analysed entirely independently, so that one array can measure two different samples, one sample analysed in the red (Cy5) channel and one analysed in the green (Cy3) channel. This means that the probe intensity measures used are not intensity ratios but raw fluorescent intensity values. These values have been background subtracted at the feature extraction stage and logs of these intensities are used throughout unless otherwise stated. Between array normalisation is not necessary since the data on different arrays, or indeed different channels, are never used in conjunction with each other for determining the individual serotype call for a sample.

The microarray contains several thousand oligonu-cleotide probes designed to detect a number of different entities:

1. It contains probes, referred to here as CPS probes, for 432 *cps *genes. On average there are 10 probes per gene. These probes are used for serotyping the sample.

2. It contains probes, referred to here as STID probes, designed to identify serotypes that are too closely related to be resolved by the *cps *genes alone.

3. There are further probes on the microarray for the entire genome of *Streptococcus pneumoniae *from two sequenced strains of the bacterium (SpTIGR4 and R6), 6824 probes in total, as well as probes for antibiotic resistance genes and for other pathogens commonly found in nasopharyngeal swabs.

The serotype analysis only uses the first two types of probes directly, the CPS probes and the STID probes, for calculating the probabilities of combinations of serotypes. Some of the other probes are used indirectly, in that the median of the log intensities of the 6824 probes for the entire *S. pneumoniae *genome is used to derive priors for the Bayes calculations in the analysis.

**Figure 1:**
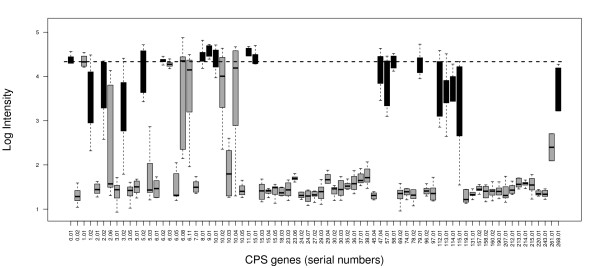
**Boxplot of CPS probe log intensities for an array testing a sample containing one serotype**. Plot of the log probe intensities for the *cps *genes from an example of a *Streptococcus pneumoniae *serotyping microarray experiment testing a sample containing only one serotype. The serial numbers of the genes are given on the horizontal axis. Only the top 75 genes (out of a total of 432 genes), with the highest mean probe log intensity are plotted for clarity. The *cps *genes found in the particular serotype being tested by this microarray experiment are marked in black. Note that some of the *cps *genes not expected in this serotype have elevated intensities. This reflects cross-hybridization of probes. The horizontal dotted line marks the median log intensity of the 6824 *S. pneumoniae *SpTIGR4+R6 genome probes.

Figure 1 shows a typical boxplot of the CPS probe log intensities from a microarray testing a sample containing one serotype. The median of the log intensities of the 6824 probes on the array for the entire *S. pneumoniae *SpTIGR4+R6 genome is also marked on the figure, as a horizontal dotted line.

Any particular serotype of *S. pneumoniae *only contains a small subset of the 432 *cps *genes. In Figure 1 the subset of *cps *genes found in the serotype being tested by this example microarray experiment are marked in black. Some of the *cps *genes *not *found in this serotype have elevated intensities. This reflects cross-hybridization of probes; in some cases it is difficult to design probes which are entirely specific to a particular gene, so probes for one gene may bind to the DNA from a different gene.

For the 91 serotypes of *S. pneumoniae *that have been identified to date, the subsets of *cps *genes that they contain are known [5,9]. As an example, the *cps *gene composition for a selection of nine of the 91 known serotypes are shown in table 1. The number of *cps *genes present in a serotype varies from as few as 1 to as many as 22, with an average of 13. In general any two serotypes will have some *cps *genes in common. And some serotypes may have very similar sets of characteristic *cps *genes, which makes differentiating between them more difficult.

**Table 1:** *Cps *gene composition of serotypes

Serotype																	
35A	0.01	1.01	2.01	3.01	5.01	16.01	45.01	128.01	129.01	130.02	131.01	51.01	132.01	7.01	21.01	6.01	18.01

35C	0.01	1.01	2.01	3.01	5.01	16.01	45.01	128.01	129.01	130.01	131.01	51.01	132.01	7.01	21.01	6.01	18.01

42	0.01	1.01	2.01	3.01	5.01	16.01	45.01	128.01	129.01	130.01	131.01	51.01	132.01	7.01	21.01	6.01	18.01

35B	0.01	1.01	2.01	3.01	5.01	16.01	45.04	129.03	117.02	51.04	7.16	21.02	6.02				

35F	0.01	1.01	2.02	3.02	61.01	16.03	45.02	46.01	24.01	32.01	7.08	21.02	6.02				

36	0.01	1.02	2.02	3.02	5.02	46.03	33.04	66.02	23.05	25.07	7.17	254.01	6.04	51.05			

38	6.04	2.03	3.04	0.02	1.03	29.02	170.01	171.01	172.01	173.01	174.01	187.01	176.01	177.01	96.01	20.03	

39	0.01	1.02	2.04	3.05	61.01	16.04	149.02	44.03	24.06	32.04	25.08	67.02	102.02	7.18	6.04	80.01	

4	0.01	1.01	2.02	3.02	261.01	29.04	35.02	212.01	30.03	213.01	214.01	215.01	23.06	36.01	37.01	38.01	

The *cps *gene composition for a selection of nine of the 91 known serotypes of *Streptococcus pneumoniae *[5]. The entries in the table are serial numbers for individual *cps *genes.

**Figure 2:**
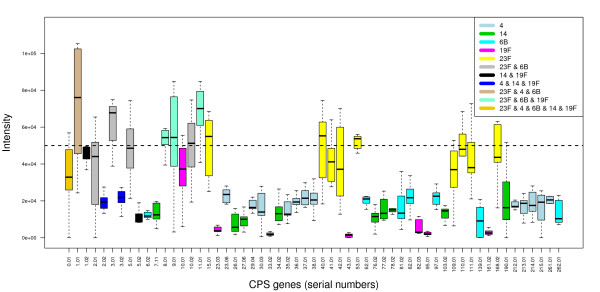
**Boxplot of CPS probe intensities for an array testing a sample containing five serotypes**. Plot of the probe intensities for the *cps *genes from an example of a *Streptococcus pneumoniae *serotyping microarray experiment testing a sample containing five serotypes. The serial numbers of the genes are given on the horizontal axis. Only the *cps *genes found in the five serotypes are plotted for clarity. The horizontal dotted line marks the median log intensity of the 6824 *S. pneumoniae *SpTIGR4+R6 genome probes. The genes are colour-coded to indicate in which serotype or serotypes they occur.

In practice a clinical sample may contain more than one serotype. Figure 2 shows a boxplot of the CPS probe intensities from a microarray testing a sample containing five serotypes, 23F, 4, 6B, 14 and 19F, in proportions 50%, 25%, 15%, 8% and 2%. Only the *cps *genes found in the five serotypes are shown in the figure for clarity. It can be seen that some of the *cps *genes are found in two or more of the serotypes contained in this sample. One gene (with serial number 0.01) is found in all five serotypes. The effect of cross-hybridisation can also be seen in Figure 2.

A further complexity in the design of the *S. pneumoniae *molecular serotyping microarray is that eighteen sets of closely related serotypes have identical, or nearly identical, sets of *cps *genes. An example can be seen in table 1 where serotypes 35C and 42 have identical gene complements, so could not be distinguished by CPS probes alone. For such cases, the microarray contains extra probes in order to distinguish between the serotypes. The extra probes are here referred to as STIDs. The serotypes that were targeted by the STID probes are listed in the first column of table 2. As can be seen from the table, most STIDs are designed to differentiate between two serotypes. Such STID probes for a pair of serotypes come in pairs, with one probe for one serotype and a paired probe for the corresponding region of the genome of the second serotype. A STID test for a pair of serotypes comprises, on average, 65 pairs of STID probes.

**Table 2:** STID tests

STID tests	Actual STID tests
11A_vs_11D	11A_vs_11D+11F

11B_vs_11C	11B_vs_11C+11A+11D

12A+12B_vs_46	12A+12B+12F+44_vs_46

12A+46_vs_12B	12A+46_vs_12B+12F+44

12F_vs_44	12F+12A+12B+46_vs_44

15B_vs_15C	15B_vs_15C

18B_vs_18C	18B_vs_18C+18A+18F

22A_vs_22F	22A_vs_22F

25A_vs_25F	25A_vs_25F

28A_vs_28F	28A_vs_28F

32A_vs_32F	32A_vs_32F

33A_vs_33F	33A+37_vs_33F

35C_vs_42	35C+35A_vs_42

40_vs_7B	40+7F_vs_7B+7C

7A_vs_7F	7A_vs_7F

9A_vs_9V	9A_vs_9V+9L+9N

9L_vs_9N	9L+9A+9V_vs_9N

6A_vs_6B	6A+6C_vs_6B+6D

The STID tests designed to discriminate identical or very similar serotypes and the actual STID tests carried out in practice.

Whilst the STIDs are designed to discriminate specific pairs of serotypes, in some cases further serotypes, closely related to the pair in question, will also elevate the intensities of the STID probes. This is because in the region of the genome being targeted by the STID probes, they have identical, or nearly identical sequences, as one or other of the pair of serotypes in question. Hence in practice the STID tests are more complex than indicated in the first column of table 2. The actual tests being carried out, due to this effect, are listed in the second column of table 2.

### Datasets used in study

We used two different experimental datasets in order to validate the probabilistic model described in this article:

1. *Reference Arrays*: 91 microarrays each testing a sample containing a single serotype.

2. *Spike-in Experiments*: four arrays with samples of more than one serotype. The composition of the combinations are given in table 3.

**Table 3:** Spike-in experiment

Sample	Serotypes	% Abundance	Estimated % Abundance
1	19F, 18C, 9V	33, 33, 33	25(10, 39), 36(23, 50), 39(28, 49)

2	19F, 18C, 9V	60, 30, 10	40(25, 55), 38(24, 53), 22(9, 34)

3	23F, 4, 6B, 14, 19F	20, 20, 20, 20, 20	26(14, 39), 12(1, 23), 21(8, 33), 25(13, 37), 16(3, 29)

4	23F, 4, 6B, 14, 19F	50, 25, 15, 8, 2	49(34, 64), 17(4, 29), 15(3, 29), 14(0, 26), 5(0, 20)

The serotype combinations and percentage abundances used in the spike-in experiment and the results of the data analysis. The figures in brackets following the estimated % abundance *π_i _*are the lower and upper 95% credible intervals.

### The likelihood of a combination of serotypes

The aim is to identify the serotypes present in a sample. The presence or absence of each of the *s *serotypes is indicated by a binary random variable *S_j _*∈ {0,1}, 1≤ *j *≤ *s*. Where *s *is the number of known serotypes, currently 91. We combine the variables in a binary vector *S *= (*S*_1_,...,*S_s_*). Depending on the context, *S *will also denote the set of indices of present serotypes. Similarly, whether any gene *i *of the *n *genes binds successfully to its CPS probes is indicated by binary random variables *G_i_*, 1≤ *i *≤ *n*. Where *n *is the number of *cps *genes, currently 432. *G *denotes the binary vector as well as the set of indices of binding genes. Each serotype *j *is associated with a subset of *cps *genes known in advance to characterize this particular serotype (for an example see table 1). We denote this set of genes by . Similarly, we denote the set of all the serotypes that contain gene *i *by .

The likelihood of the combination of serotypes consists of three parts enumerated below:

1. *Likelihood of a gene binding depending on CPS probes*. We denote the set of log intensities of CPS probes for *cps *gene *i *by *y_i_*, 1≤ *i *≤ *n*. The vector *y *= (*y*_1_,...,*y_n_*) denotes the data sets for all the *cps *genes on the microarray. This part describes the likelihood of gene *i *binding depending on its probes *y_i_*.

2. *Likelihood of a serotype combination depending on gene binding*. Each serotype is defined by a characteristic subset of *cps *genes. The subsets of *cps *genes of different serotypes might partly be identical. In addition genes occurring in the characteristic subsets of genes of two different serotypes may be very similar (homologous) to each other, resulting in cross-hybridisation. These dependencies between serotypes need to be taken into account. This part describes the likelihood of a serotype combination *S *depending on genes binding and the grouping of genes according to homology.

3. *Likelihood of a serotype combination depending on STID probes*. In order to help with the differentiation between serotypes with identical or very similar sets of *cps *genes, additional probes are added to the microarrays, the STID probes. Each set *d_l_*, 1 ≤ *l *≤ *L*, of STID probes is designed to differentiate between two serotypes (or more generally, two groups of serotypes). The vector *d *= (*d*_1_,...,*d_L_*) denotes all the STID data from a microarray experiment. This part describes the likelihood of a serotype combination *S *depending on STID probes *d*.

These three likelihoods are explained in the following three sections. The fourth section, combines the above likelihoods into *P*(*y*, *d *|*S*) for a serotype combination *S *and data *y*, *d*. This likelihood can be evaluated for any combination *S *of the 91 serotypes. Since this is impractical for all 2^91 ^possible combinations, we resort to using a heuristic to select a subset of combinations of serotypes. We also suggest a prior *P*(*S*) in this section.

The prior *P*(*S*) and likelihood *P*(*y*, *d *|*S*) for *S *allow us to calculated the posterior *P*(*S *|*y*, *d*) apart from a normalising constant

#### Likelihood of a gene binding depending on CPS probes

Each *cps *gene *i *is represented by a set of about ten CPS probes with log intensities , where *r_i _*is the number of probes on the array for gene *i*. Assume the true log intensity value for binding of a gene *i *is *μ_i_*, with . We simplify notation by setting . If *G_i _*= 1, that is, binding is successful, then *μ_i _*>*m*, where *m *is the log intensity of the background signal due to unspecific binding. If *G_i _*= 0, that is, binding is unsuccessful, then *μ_i _*= *m*. Since we have little information about *μ_i_*, *m*, and  we consider them to be nuisance parameters over which should be integrated. Assuming reasonable priors for these parameters we can calculate *P*(*y_i _*| *G_i _*= 0) and *P*(*y_i _*| *G_i _*= 1) as follows.

**Figure 3:**
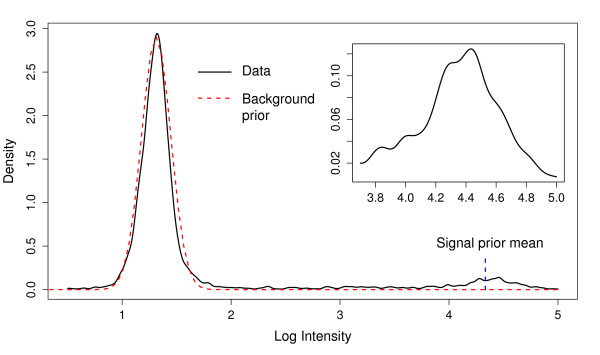
**Density estimate of CPS probe intensities**. Density estimate of the log probe intensities for 432 *cps *genes from a *Streptococcus pneumoniae *serotyping microarray testing strain 24A. The background prior distribution  and the value of the signal prior mean *μ*_(1) _for this data are also shown. The inset shows the signal part of the distribution in more detail.

In general, visual inspection of distributions of log intensities suggests that assumptions of Gaussian distributions on the above parameters might not oversimplify matters too much (see Figure 3 for an example of a typical distribution). This is also the standard assumption in much of the analysis of log intensity values from microarray experiments in the literature [10].

As priors for *μ_i_*, *m*, and  we assume:

1. , a scaled inverse *χ*^2 ^distribution with shape and scale parameter *ν*_0 _and  (for details see Additional file 1).

2. , where *μ*_0 _and  are the prior mean and variance of the background distribution of log intensity values.

3. *μ*_*i *_= *m *if *G_i _*= 0, and  and *μ_i _*>*m *if *G_i _*= 1, where *μ*_(1) _is the prior mean for the log intensity signal of the probes when a gene binds, and *κ*_1 _is a prior scaling factor for the variance.

For *G_i _*= 0 we obtain the likelihood(1)

Here *p*_1_(*y_i_*|*μ*, *ν*_0_, ) is a multivariate *t *distribution as in equation 2 in Additional file 1. The integral over *μ *is solved numerically. Similarly, for *G_i _*= 1 we obtain(2)

where *I *is an indicator variable for an event, and *p*_2_(*y_i_*, *μ*|μ_(1) _*κ*_1_, *ν*_0_, *ρ*_0_) is a multivariate *t *distribution as in equation 4 in Additional file 1. The integral over *μ *is solved numerically: the 'int' function of the R [11] package 'rmutil' [12] was found to be a reliable option for evaluating these equations with the given data. Equation 2 is normalised by a factor obtained by a similar numerical integration where the term *p_N_*(*y_i_*|*μ*, *σ*^2^) is dropped.

Constants for the prior distributions were chosen using statistics from the arrays, so that the above approach is a test of hypotheses via an empirical Bayes procedure:

1. For the Gaussian prior on background signal  we set the mean *μ*_0 _to the mode of all the log intensities on the array. The scale parameter  was set to the 0.33 quantile of all log intensities below the mode. This seems to capture the overall distribution of unspecific signals. Figure 3 shows the density distribution of the data plotted in Figure 1, with the background prior distribution  superimposed.

2. The prior mean *μ*_(1) _for the log signal intensity was set to the median of the data from the 6824 probes on the array for the entire *S. pneumoniae *SpTIGR4+R6 genome. This genome provides a standard reference level for a binding signal, but is actually independent of the binding of the *cps *genes in our model. The value of the prior mean for the log signal intensity *μ*_(1) _is marked on Figure 3.

Two values were used for the prior scaling factor for the variance *κ*_1_. For increased specificity, that is, less chance of false positives, but with a concomitant increase in false negatives, a value of *κ*_1 _= 13 was used, which is the average number of genes of a serotype. For increased sensitivity, that is, fewer false negatives, but possibly more false positives, a value of *κ*_1 _= 1 was used.

3. The shape parameter *ν*_0 _of the variance prior  was set to 1 in all cases to give a reasonably broad prior distribution on the noise variance. The scale parameter *ρ*_0 _of the variance prior is calculated as 1/4 of the distance between *μ*_0 _and *μ*_(1)_.

### Likelihood of a serotype combination depending on gene binding

If any one of the serotypes  containing gene *i *is present, we expect *G_i _*= 1, that is, gene *i *binds to its probes. We assume that the binding might fail with small probability *β *for one serotype. The reason may be experimental failure or biological variation. For simplicity we further assume that these failures are independent for each serotype. Hence the probability that a gene *i *fails to bind is *β^k^*, where  is the number of serotypes present in the sample and containing gene *i*. On the other hand, there might be a small probability *α *of a spurious binding response. After consultation with experimentalists, these parameters are assumed to be around *α *= *β *= 0.01 (see the results section for maximum likelihood estimations of these values).

Some of the *cps *genes are evolutionarily closely related (homologous) [6,7]. Their DNA sequences may be quite similar. The intensities of the probes for a particular gene may be elevated not by the presence of the gene they were designed to target, but by the presence of a homologous gene. The probes on the array have been designed to minimize cross-hybridisation, and in practice we find that the signal for most genes are independent. There is however a subset of 30 genes in 9 different homology groups where there is significant cross-hybridisation. We consider these 30 genes as belonging to 9 cross-hybridisation groups, and the remaining 402 genes as belonging to 402 groups containing just one gene. We assume a constant probability *γ *that a gene wrongly appears as present due to the presence of another gene in the same cross-hybridisation group. After consultation with experimentalists we set the value of *γ *to 0.95. A maximum likelihood estimation of *γ *is presented in the results section.

For the sake of brevity denote the set of genes present in a set of serotypes by . The binary variable *H_k _*indicates the presence of a representative gene of cross-hybridisation group *k *in . ℋ(*k*) is the index set of genes belonging to group *k*. When a serotype is present with a gene from the hybridisation group, that group counts as present:(3)

For *i *∈ ℋ(*k*) we have(4)

Note that in the third condition *H_k _*= 0 means that no member of the cross-hybridisation group ℋ(*k*) is present, in contradiction to gene *i *being a member (*i *∈ ℋ(*k*)) and being present ; the corresponding probability can be set to any arbitrary value, say 0.

#### Likelihood of a serotype combination depending on STID probes

A number of pairs of serotypes have identical *cps *gene complements. In order to distinguish between these serotypes the microarray contains extra oligonucleotide probes which test subtle genetic differences between key *cps *genes of these serotypes. These extra probes are referred to as STIDs.

STID probes are different from the CPS probes in that they enable a direct comparison of two specific sets of serotypes: a set *T_l _*of STID probes is designed to show the presence or absence of any serotype of a set  of serotypes compared to presence or absence of any serotype of another set  of serotypes. The STID probes for  and  are paired, that is, each probe for one serotype set has a corresponding probe for the other serotype set. We analyse the difference of values of the paired probes. For a set *T_l _*of STIDs we denote the measured differences in log intensities by *d_l_*. Similar to equations 1 and 2 we define

where  and  are as defined in equations 1 and 2; they can be expressed analytically as in equation 2 and 4 in Additional file 1. We obtain for the distribution of differences between log intensities of STID probes(5)

After inspection of typical differences in log intensities of STID probes we use prior values *μ*_2 _= 0.5,  =  = *ν*_2 _= *κ*_2 _= 1

#### Posterior of combinations of serotypes

The likelihood of a given serotype combination is given by combining equations 1, 2, 3, 4, and 5

**Figure 4:**
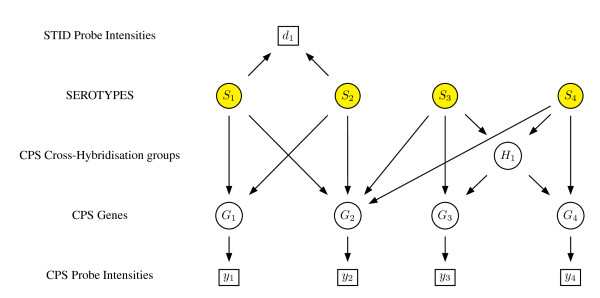
**An example of a model for serotype combinations showing independence relationships as a graphical model**. The binary variable *S_i _*indicates the presence/absence of serotype *i*, the binary variable *G_j _*that of gene *j*, and *y_j _*a set of log intensities from the CPS probes representing gene *j*. Binary variable *H_k _*indicates the presence of a representative gene of cross-hybridisation group *k*. *d_l _*indicates a set of log intensities from the STID probes (square boxes indicate data).

This model is represented schematically in Figure 4.

In practice there are too many different serotypes *S*, currently 91, to calculate *P *(*y*, *d *| *S*) for all possible 2^91 ^combinations. The question is whether we need to test all possible combinations of serotypes. In principle some higher order combination of serotypes might show an unexpectedly high probability, higher than any of its subsets, for example, due to the cross hybridisation effect. However, here we assume that such cases are rare. The observation that in clinical samples a combination containing more than three serotypes is highly unusual provides a further justification for this assumption.

Therefore we try to catch at least all pairwise interactions by calculating *P *(*y *| *S*) for all *S *with at most two serotypes. We assume it is unlikely that higher order interactions are not visible in at least pairwise interactions. The serotypes from the thirty most probable of these pairwise combinations are pooled and used to calculate *P *(*y *| *S*) for all possible combinations of three or fewer serotypes out of the pool, thus allowing for some high probability three-way interactions that have less probable two-way interactions. Finally, serotypes of the fifteen most probable combinations from this pool are selected to create a final pool of serotypes that is small enough to allow calculation of the probability of combinations of eight or less serotypes from the pool.

In order to calculate the posterior *P *(S | *y*, *d*) from the likelihood we require a prior *P *(*S*). Let *P*(*λ*) denote the prior probability of being infected by a number of serotypes *λ *out of a total of *s *= 91 possible serotypes. Since there are  serotype combinations with *λ *serotypes this implies a prior *P*(*S*) of

where *λ*(*S*) is the number of serotypes in *S*. The probability *P*(*λ*) is probably declining rapidly with *λ*. Since the exact distribution is unknown, we assume for simplicity that this probability is constant *P*(*λ*) = 1/(*s *+ 1) for all *λ *(including the possibility that no serotype is present). Prior and likelihood for *S *allow us to calculated the posterior *P*(*S *| *y*, *d*) apart from a normalising constant

The software implementation of the analysis method normalises *P*(*y*, *d *| *S*)*P*(*S*) for a particular set of combinations of serotypes *S*, using the sum of *P*(*y*, *d *| *S*)*P*(*S*) from all the combinations of serotypes chosen according to the above selection heuristic. Since these combinations seem to capture most of the posterior probability mass for *S *we take these normalised values as approximation to the posterior *P*(*S *| *y*, *d*).

### Proportional abundance of serotypes

If the analysis detects the presence of more than one serotype within a sample then information on the relative abundance of the serotypes is desirable. Each serotype is represented by a selection of genes, whose abundance can be measured. This suggests an ANOVA analysis to estimate abundance of serotypes. Again we opt for a Bayesian treatment which allows us to integrate out nuisance variables such as gene specific effects and to derive credible intervals for the serotype abundance estimates.

For the following analysis we work with raw intensities without log transformation since serotype presence and abundance will have an additive effect on gene specific intensities. For obtaining an analytical solution, a linear model with untruncated Gaussian distributions as error and prior distributions is convenient. Since intensities can only be positive such a model can only be considered an approximation. A further problem is that the variance of a variable measuring abundance is often not constant and depends on the size of the variable. This is mitigated to some degree by integrating over variances in the Bayesian model. Despite its shortcomings, the results indicate the model reproduces experimental data reasonably well.

More specifically, we want to estimate the proportion of *b *serotypes in the sample. Each serotype is represented by a set of genes as specified in a *g *× *b *binary 0/1 matrix *G*, where *g *is the number of genes involved. We assume that gene abundance as reflected in its probe intensities is linearly related to the sum of the abundances of serotypes containing the gene. Each gene in turn is represented by a set of probes. From visual inspection (Figure 1) it is clear that all these probes are affected in a similar way by gene specific noise. An *n *× *g *binary 0/1 matrix *Z *indicates which of the *n *probes represents each of the *g *genes and a vector *u *represents gene specific noise levels. The information in the matrices *G *and *Z *can be combined in a *n *× *b *binary 0/1 matrix *X *indicating for each of the *b *serotypes by which of the n probes it is represented.

The response variables *w_j _*≥ 0, 1 ≤ *j *≤ *n*, are intensities of the probes. A random effects ANOVA model is

Where *θ *is a *b*-vector of the abundance of serotypes, *u *is a *g*-vector of nuisance parameters for noise affecting all probes of a gene in the same way, and *ε *is a noise term for individual probes. Note that there is no mean parameter and the entries in the matrix *X *are 0 or 1. Hence *θ *is the vector of direct (nonnegative) abundances of all serotypes in the sample. As priors we assume , , and , where *A *is a *b *× *b *matrix, *B *is a *g *× *g *matrix, *I_n _*is the unit matrix, and *c_B _*and *c_A _*are scaling constants. The matrices *A *and *B *are fixed in advance, while the scaling constants *c_B _*and *c_A _*are considered hyperparameters. The matrix *B *is set to *B *= *G'G*, that is, the more genes two serotypes have in common the higher their abundance is assumed to correlate. Matrix *A *is simply set to *I_g_*. To enable the calculation of an analytical solution for the posterior *θ *of via a conjugate analysis and also to make the scaling constants *c_A _*and *c_B _*identifiable, we assume . An inverse *χ*^2 ^prior with degree *ν*_0 _= 10 and expected variance  is assumed for . Detailed derivations for this model are provided in Additional file 2. Analysis of simulated data showed that the hyperparameters *c_A_*, *c_B_*, *ν*_0 _and  are best set in an empirical Bayes fashion by optimising the marginal likelihood .

Once a posterior distribution (a multivariate *t *distribution, see Additional file 2) with mean  for the serotype abundances *θ *is derived, an estimate of the proportions of serotypes is provided by . Due to our model assumptions  can be negative, in which case it is reset to 0. To obtain 95% credible intervals for *π_i _*we draw 10000 samples *θ*^(*k*) ^from the posterior of *θ *and obtain sample proportions . Credible (marginal) intervals for *π_i _*are then easily obtained from the distributions of simulated serotype proportions .

## Results and Discussion

### Reference arrays

The analysis method gave a correct result for 88 of the 91 reference arrays. Prior parameter values were *κ*_1 _= 13, *α *= 0.01, *β *= 0.01, *γ *= 0.95. Three reference arrays, testing serotypes 44, 25A and 37, appeared to give incorrect results.

On the reference array testing serotype 44 the analysis called serotype 12F as present, and on the array testing 25A, serotype 25F was called as present. These are two pairs of closely related serotypes and therefore it is likely that the probes designed to discriminate these were not performing optimally or were targeting a poor region for reliable differentiation. The array testing serotype 37 detected a combination of serotype 37 and serotype 33A. This is expected since serotype 37 contains a non-functional copy of the *cps *gene complement of serotype 33A. Hence the Bayesian model does produce the correct call after all.

**Figure 5:**
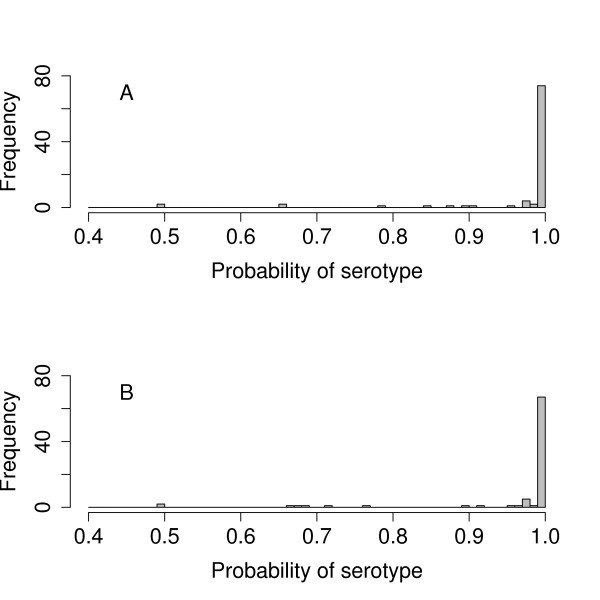
**Reference arrays results**. Histogram of the highest probability *P*(*S*|*y*, *d*) in the analysis of each of the 91 reference arrays for *κ*_1 _= 13 (A) and *κ*_1 _= 1 (B)

For the majority of the reference arrays the particular serotype being tested in the array experiment had a probability *P*(*S *| *y*, *d*) of effectively 1, the other 90 serotypes having extremely low probabilities. For a few arrays the serotype being tested had a probability markedly lower than 1. Figure 5A shows a histogram of the highest probability *P*(*S *| *y*, *d*) in the analysis of each of the 91 reference arrays. The lower probabilities of a few of the correct serotypes are due to the existence of another serotype with a very similar *cps *gene complement, differing by just one gene.

Originally the model did not include cross-hybridisation. Without the inclusion of cross-hybridisation the model still gives relatively good results, although three more reference arrays gave incorrect calls. The array testing serotype 24F called a combination of 24F and 24B, whilst the array testing 33A called 33F, and the array testing 7B called a combination of 7B and 7C. The inclusion of cross-hybridisation solved these three problem serotypes.

### Spike-in experiment

Analysis of the four samples in the spike-in experiment gave the correct serotype combinations, with no false positives and no false negatives. The parameter values, *κ*_1 _= 13, *α *= 0.01, *β *= 0.01, *γ *= 0.95, were the same as used for the analysis of the reference arrays.

**Figure 6:**
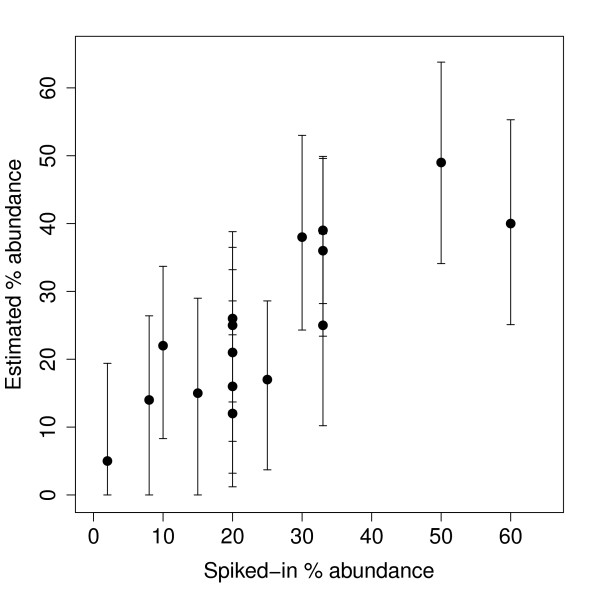
**Spike-in arrays results**. Percentage abundance estimated by the analysis plotted against the actual spiked-in percentage abundance, from the spike-in experiment.

Table 3 gives the results for the spike-in experiment and Figure 6 plots the measured percentage abundance against the spiked-in percentage abundance. The estimates of the percentage abundance of the serotypes in the combinations agreed reasonably well with the actual spiked-in percentages. The only exception is sample 2 in table 3 where the estimate of the first component is slightly too low. However, spike-in experiments with exact amounts of the pathogen are difficult to perform; that is the target numbers in the third column of table 3 are approximate only.

### Influence of prior value *κ*_1_

The results for the reference arrays in Figure 5A are for a value of *κ*_1 _= 13, which gives optimal results. In a clinical context, however, not missing a serotype present in a sample in only trace abundance is of importance. We therefore provide a second choice of a high sensitivity setting with *κ*_1 _= 1 in the software implementation of the analysis. Figure 5B shows a histogram of the highest *P*(*S *| *y*, *d*) probability in the analysis of each of the 91 reference array experiments when *κ*_1 _= 1. When *κ*_1 _= 1 there are three extra apparent errors.

The array calling serotype 23F calls a combination of serotypes 23F and 23B. These two serotypes have thirteen *cps *genes in common and five different. The five differences are in *cps *genes which are closely related. The extra sensitivity of the analysis with *κ*_1 _= 1 is detecting the five *cps *genes of serotype 23B. These have levels slightly elevated from the background due in part to cross-hybridisation from the corresponding five genes in serotype 23F. Refinement of the cross-hybridisation analysis may be able to resolve this problem, although the fact that 23F and 23B are closely related means that from a clinical stand point this false positive is not too critical. In addition the software implementation of the analysis carries out further checks. For each called serotype, the software checks the probabilities of that serotype's genes (*P*(*y *| *G_i _*= 1)). If any serotype's genes have *P*(*y *| *G_i _*= 1) < 0.5 that serotype is flagged as such. In this case serotype 23B is flagged as having genes with *P*(*y *| *G_i _*= 1) < 0.5, alerting users to the need for further investigation.

The array testing serotype 33A now calls a combination of serotypes 33A & 29, and the array testing serotype 14 calls a combination of 14 & 19A. Serotypes 33A and 29 are not closely related, with only one *cps *gene in common. Similarly serotypes 14 and 19A are also unrelated with only one *cps *gene in common, so it was thought unlikely that these incorrect calls were due to a problem with the analysis. Further analysis indicates that the genes for 29 and 19A are actually present in very low relative abundance in the samples and we think that these calls are due to contamination of samples 33A and 14 at some stage in the experimental process. Eight microarrays are mounted on a single glass slide. The arrays testing serotypes 14 and 19A were adjacent on the glass slide so contamination of the sample containing serotype 14 with serotype 19A at this stage of the experiment is plausible. If the assumption of contamination is correct, the setting of *κ*_1 _= 1 seems to be able to detect the presence of contaminating serotypes at very low abundance levels.

For the spike-in experiment, with a value of *κ*_1 _= 1 the analysis still identified all the correct serotypes as being present but, due to the higher sensitivity at this set-ting, the analysis also gave some false positives (Sample 1: 19A, Sample 2: 19A, Sample 3: 19A, Sample 4: 23B). All the false positives were flagged by the software as having genes with *P*(*y *| *G_i _*= 1) < 0.5, but none of the true positives were flagged.

We recommend that users of the software implementation of the algorithm run the analysis twice. An initial run with *κ*_1 _= 13 will indicate the main serotype or serotypes present in the sample. A second run with *κ*_1 _= 1 will indicate if there may also be extra low abundance serotypes present. If any of these serotypes have genes with *P*(*y *| *G_i _*= 1) < 0.5 they will be flagged, to alert the user that they may warrant further investigation.

### Influence of prior values *α*, *β *and *γ*

**Figure 7:**
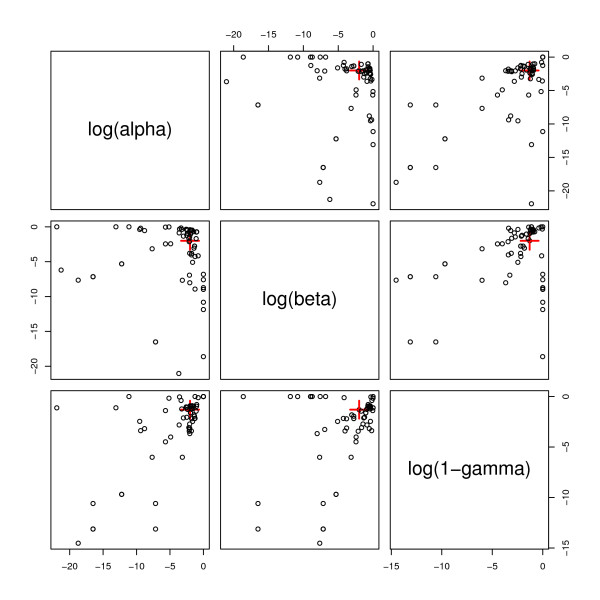
**Optimum values of *α*, *β *and 1 -*γ***. Pairwise plots of the optimum values (log10) of *α*, *β *and 1 -*γ*. Each point represents the optimum values of *α*, *β *and 1 -*γ *for one of the reference arrays. The crosses mark the positions of the actual values of *α*, *β *and 1 -*γ *used in the analysis (*α *= 0.01, *β *= 0.01 and 1 -*γ *= 0.05).

The optimum values of *α*, *β *and *γ *will vary with serotype being studied, so general values of the three priors that work well for all serotypes were chosen, based on expert estimates of expected true and false response rates of probes, namely *α *= 0.01, *β *= 0.01, *γ *= 0.95. The suitability of the chosen values was investigated further. Each reference array was analysed in turn. The values of *α*, *β *and *γ *that gave the highest value of *P*(*S_i _*| *y*) for the serotype that the array was testing were found by numerical optimisation. Figure 7 shows pairwise plots of the optimum values for *α*, *β *and *γ *for the 91 reference arrays. Whilst there are some outliers the optimum values for *α*, *β *and *γ *for most serotypes cluster in the region of the chosen values *α *= 0.01, *β *= 0.01, 1 -*γ *= 0.05.

The sensitivity of the results to the values of *α*, *β *and *γ *was also investigated. The 91 reference arrays were analysed in turn. Each array was analysed with all combinations of eight different values of *α *and *β *(0, 0.001, 0.005, 0.01, 0.02, 0.05, 0.1, 0.3) and *γ *(1, 0.99, 0.975, 0.95, 0.9, 0.8, 0.7, 0.5). The effect of the prior values that we are most interested in is not so much the absolute value of *P*(*S *| *y*, *d*) for the serotype *S *being tested on the array, but whether this probability is the highest on that array. Therefore for each combination of prior values the fraction of the 91 reference arrays that call the correct serotype as the most probable serotype on the array was recorded.

**Figure 8:**
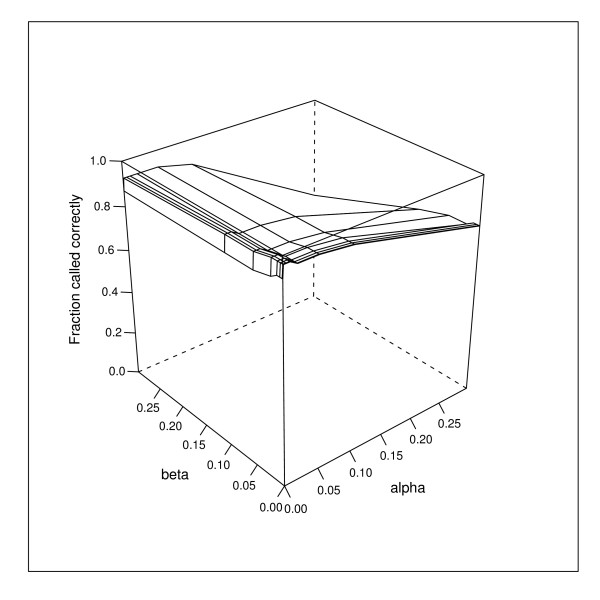
**Influence of *α *and *β *on fraction of correctly called serotypes**. Surface plot showing how the fraction of correctly called serotypes in the reference array data set varies with the values of priors *α *and *β*.

The results for *α *and *β *are presented in Figure 8. The figure shows how the fraction of correctly called serotypes varies with *α *and *β *(*γ *being held at its default value of 0.95). The default values of *α *= 0.01 and *β *= 0.01 lie within the optimal range to generate the maximum number of correctly called serotypes.

It can be seen from Figure 8 that values of *β *between 0 and 0.02 give the same results as the default of *β *= 0.01. *β *is the false negative rate for binding, allowing for those genes that do not bind to their probes, despite a serotype that contains those genes being present in a sample. For the reference arrays, which test samples containing only one serotype, the intensities of the probes that should be present are sufficiently high for this correction to be less important. The benefit of including *β *= 0.01, arises when a sample contains a combination of serotypes, some with low percentage abundance. Then the intensities of some of the probes that should be present will be much closer to the background noise (see Figure 2).

Whereas the value of *γ *effects the value of *P*(*S_i _*| *y*), the correct calling of the three serotypes that are influenced by cross-hybridisation (24F, 33A and 7B) was found to be insensitive to the values of *γ *tested. As the serotyping array develops in the future, as more closely related serotypes of *S. pneumoniae *are discovered, and more probes are added to the array, then cross-hybridisation may become a greater problem and it may be necessary to adjust the value of *γ *to give optimal results.

## Conclusions

The *Streptococcus pneumoniae *molecular serotyping microarray combined with an empirical Bayesian data analysis presents two main advantages over conventional methods for serotyping strains. Firstly, it is extremely accurate in identifying the correct serotype in single serotype samples. Secondly, it has the ability to easily detect combinations of serotypes within a sample. Initially we tried a simpler analysis of the array data, based on frequentist methods, in which *p*-values for individual genes were calculated using *t*-tests and then combined. On the reference arrays a *p*-value approach gave thirteen incorrect serotype calls, an unacceptably high error rate, so the current Bayesian model was adopted. The empirical Bayesian data analysis does give two incorrect serotype calls for closely related pairs of serotypes, but these errors have been identified as design problems with the array rather than a problem with the analysis, a de-sign problem that will be addressed in future releases of the array.

The Bayesian approach enables additional information, such as on cross-hybridisation or on STIDs, to be integrated into the main model. The method does not require accurate estimates of prior parameters, working well with general estimates of these values for all serotypes. A few hyperparameters are estimated from the data in an empirical Bayes fashion, but in a way that is independent of knowledge of present serotypes or combinations of serotypes. The prior parameters were chosen to be standardised and repeatable across arrays, where levels of signal or background intensity can change. For the reference arrays the signal in the data is reasonably strong. However, for the low percent-age abundance serotypes in the spike-in arrays the signal to noise ratio is much lower, but the same prior parameter values work well.

The spike-in experiment indicates that the method can detect multiple serotypes in samples with as large a number of serotypes as is ever likely to be found in a clinical setting. Serotypes with very low abundance within a combination can be detected by the method. As well as detecting the presence of serotype combinations, an approximate measure of the percentage abundance of the serotypes within the combination can be obtained. Of the 16 estimated abundances in table 3, one serotype (19F in sample 2) lies slightly outside the credible interval. Further experimental work will be required to determine whether this is a problem with the statistical analysis or has arisen from experimental imprecision in the creation of this particular spike-in sample. However the overall conclusions from the results to date suggest that as well as the molecular serotyping microarray's primary role as a method for calling serotypes, it can also provide a useful indication of serotype relative abundance.

In this article we have proposed two separate models. The first is for calculating posterior probabilities of combinations of serotypes where only presence or absence of serotypes is considered. The second model estimates the relative abundance of serotypes. In principle the two models could be combined. However, the primary clinical interest is in calling presence or absence of serotypes. Their quantitative assessment is of secondary interest. A model comprising both would be more complex and might compromise the performance as a serotype caller.

The linear Gaussian model with constant variances for estimating relative abundances of serotypes makes assumptions which are certainly only approximately correct. For example, abundances are always positive and variances will depend on the abundance values. However, in simulations and in the analysis of the spike-in data this model performed considerably better than simpler models without integration over variances or over the hidden gene abundances. That is, under the constraint of having a model that can be solved analytically, which is important when analysing larger data sets interactively as is currently done with the present software, the current model is an excellent approximation.

This algorithm has been applied to over one thousand clinical samples, containing both single serotypes and combinations of serotypes. The *cps *gene content of these samples have been checked by manual data analysis methods which has confirmed that the technique described in this article is a reliable automatic analysis method for the *S. pneumoniae *molecular serotyping microarray. The inference could be affected by the heuristic we employ to reduce the number of combinations tested to a computationally feasible level. Increasing the number of combinations tested does not produce any extra combinations of serotypes that have a non-negligible probability, when applied to the clinical data or the reference and spiked-in arrays, indicating that the current cut-off is quite generous.

In this Bayesian method to analyse the *S. pneumoniae *molecular serotyping microarray the serotype combination with the highest probability is accepted as the answer. Alternatively, probabilities for properties of interest, for example, the co-occurrence of specific serotypes, may be obtained by model averaging, that is, by summing probabilities of the corresponding serotype combinations. The method is also easily extensible as more is learned of the different strains of this important pathogen. The use of diagnostic microarrays is not confined to the field of infectious diseases. For example arrays have been used as potential diagnostic tools in oncology [13] and cytogenetics [14]. The statistical analysis method is quite general and is easily adapted for other diagnostic microarrays that use similar technology. The Bayesian approach means that if these microarrays contain extra features, they can be incorporated into the analysis with minimal modification.

An alternative solution to the problem of cross-hybridisation would be to reannotate the *cps *gene com-positions of the serotypes. The advantage of our approach is that the invariant biological input to the model, that is the *cps *gene complements of the serotypes determined experimentally by sequencing is separate from the cross-hybridisation input which is a design issue with the array and may not be invariant. The array is being constantly revised and improved so the Bayesian model keeps separate the information that will not change from the information that may change in the future.

The method as it stands works very well. Future work will concentrate on solving problems that may arise as further data sets become available. Adding prior information on gene specific variances and STID specific variances will be investigated as a method for improving the accuracy of the analysis [15]. In general cross-hybridisation is not a problem due to careful probe design. Where it is a problem the current model treats it at the gene rather than at the probe level. Dealing with cross-hybridisation at the probe level may improve the model. However, cross-hybridisation is only a problem between some of the homologous genes, which are very similar in sequence. This means that most of the genes' probes do cross-hybridise, so treating cross-hybridisation at the probe level may not significantly improve performance. An advantage of the current approach is that it is essentially analytical so works well without the need for expert input, and is not too computationally intensive. But the potential of more complex models that require a sampling approach should also be investigated.

The algorithms described in this paper were implemented in the R statistical system [11]. For the benefit of researchers in the field of *S. pneumoniae *who are unfamiliar with R, a user friendly web interface was created for the R script. This web interface was created using the web application Rwui [16].

## Authors' contributions

LW and RN devised the statistical analysis. JH contributed the experimental data and guided interpretation. All authors read and approved the final manuscript.

## Supplementary Material

Additional file 1 **Distributions and posteriors**. Equations and derivations for all distributions used in calculating the probabilities of combinations of serotypes. Click here for file

Additional file 2 **Bayesian Anova**. Equations and derivation of the Bayesian Anova. Click here for file
