# Supplementary material for: Empirical Bayesian models for analysing molecular serotyping microarrays
Source: BMC Bioinformatics. 2011 Mar 31;12:88. doi: 10.1186/1471-2105-12-88 (PMC3076268; doi:10.1186/1471-2105-12-88)
Supplement: Additional file 1 — Distributions and posteriors. Equations and derivations for all distributions used in calculating the probabilities of combinations of serotypes. [file 1471-2105-12-88-S1.PDF]

Appendix 1: Distributions and posteriors

Richard Newton*[1], Jason Hinds[2] and Lorenz Wernisch[1]

[1]MRC Biostatistics Unit, Robinson Way, Cambridge, CB2 0SR, UK
[2]Bacterial Microarray Group, Division of Cellular & Molecular Medicine,St. George's, University of London, Cranmer Terrace, London, SW17 0RE, UK

Email: Richard Newton*- richard.newton@mrc-bsu.cam.ac.uk; Jason Hinds - j.hinds@sgul.ac.uk ; Lorenz Wernisch - lorenz.wernisch@mrc-bsu.cam.ac.uk;

*Corresponding author

Although they are standard, for completeness and the convenience of the reader interested in the details of the calculations we provide equations and derivations for all distributions used in the paper. As a prior on the variance we use the scaled Inv-$\chi^2(\nu_0, \sigma_0^2) = \text{Inv-Gamma}(\nu_0/2, \nu_0\sigma_0^2/2)$ distribution

$$p_{\text{ICh}}(\sigma^2 \mid \nu_0, \rho_0^2) = \frac{(\nu_0\rho_0^2/2)^{\nu_0/2}}{\Gamma(\nu_0/2)}(\sigma^2)^{-(\nu_0/2+1)}e^{-\nu_0\rho_0^2/(2\sigma^2)}$$

with shape parameter ν_0 and variance parameter ρ_0^2. A d-dimensional multivariate t-distribution with mean μ, variance $\frac{\nu}{\nu-2}\Sigma$ and degrees of freedom ν is

$$p_t(\theta \mid \mu, \nu, \Sigma) = \frac{\Gamma((\nu+d)/2)}{\Gamma(\nu/2)\nu^{d/2}\pi^{d/2}}|\Sigma|^{-1/2}(1 + \frac{1}{\nu}(\theta-\mu)'\Sigma^{-1}(\theta-\mu))$$

In this section we will make repeated use of the following integration. Assume the joint probability of some d-dimensional parameter θ and variance σ^2 is

$$p(\theta, \sigma^2 \mid \mu, m, \Sigma, s^2) \propto \frac{1}{(\sigma^2)^{(m+d)/2+1}}\exp(-\frac{(\theta-\mu)'\Sigma^{-1}(\theta-\mu)+s^2}{2\sigma^2})$$

Using the definitions of the χ^2 and of the t distribution the following integral over σ^2 is easily calculated.

$$\int_0^\infty p(\theta, \sigma^2 \mid \mu, m, \Sigma, s^2)\, d(\sigma^2) = p_t(\theta \mid \mu, m, s^2\Sigma) \tag{1}$$

Assume data $y = (y_1, \ldots, y_k)$ are distributed normally around mean μ, with variance σ^2 under a Inv-$\chi^2(\nu_0, \rho_0^2)$ prior. Integrating over the variance we obtain

$$\begin{aligned} p_1(y \mid \mu, \nu_0, \rho_0^2) &= \int p_{\text{N}}(y \mid \mu, \sigma^2) p_{\text{ICh}}(\sigma^2 \mid \nu_0, \rho_0^2)\, d(\sigma^2) \\ &\propto \int \frac{1}{(\sigma^2)^{(k+\nu_0)/2+1}}\exp(-\frac{\sum(y_l-\mu)^2+\nu_0\rho_0^2}{2\sigma^2})\, d(\sigma^2) \\ &= p_t(y \mid (\mu, \ldots, \mu), \nu_0, \nu_0\rho_0^2 I_k) \end{aligned} \tag{2}$$

The hyperparameter ν_0 has the function of a pseudocount expressing confidence in the prior on σ^2. If the joint probability of y and μ is required with a Gaussian prior on μ we have with $\mu_2 = \mu_1\kappa_1/(\kappa_1 + k - 1)$

$$\begin{aligned}
&\sum(y_l - \mu)^2 + \kappa_1(\mu - \mu_1)^2 + \nu_0\rho_0^2 \\
&\quad = \sum(y_l - \mu_2)^2 - 2\sum(y_l - \mu_2)(\mu - \mu_2) + (k + \kappa_1)(\mu - \mu_2)^2 + \frac{(k-1)\kappa_1}{k - 1 + \kappa_1}\mu_1^2
\end{aligned} \tag{3}$$

and so

$$\begin{aligned}
&p_2(y, \mu \mid \mu_1, \kappa_1, \nu_0, \rho_0^2) = \int p_{\mathrm{N}}(y \mid \mu, \sigma^2)\, p_{\mathrm{N}}(\mu \mid \mu_1, \sigma^2/\kappa_1)\, p_{\mathrm{ICh}}(\sigma^2 \mid \nu_0, \rho_0^2)\, d(\sigma^2) \\
&\quad = \int \frac{1}{(\sigma^2)^{(k+1+\nu_0)/2+1}} \exp(-\frac{\sum(y_l - \mu)^2 + \kappa_1(\mu - \mu_1)^2 + \nu_0\rho_0^2}{2\sigma^2})\, d(\sigma^2) \\
&\quad = p_t(y, \mu \mid (\mu_2, \ldots, \mu_2), \nu_0, (\frac{(k-1)\kappa_1}{k - 1 + \kappa_1}\mu_1^2 + \nu_0\rho_0^2) D_{k+1})
\end{aligned} \tag{4}$$

where D_{k+1} is the identity matrix except for the last row and column which consists of the vector $(-1, \ldots, -1, \kappa_1 + k)$. Here, κ_1 has the function of a pseudocount expressing the confidence in the prior mean μ_1.
