# Supplementary material for: Empirical Bayesian models for analysing molecular serotyping microarrays
Source: BMC Bioinformatics. 2011 Mar 31;12:88. doi: 10.1186/1471-2105-12-88 (PMC3076268; doi:10.1186/1471-2105-12-88)
Supplement: Additional file 2 — Bayesian Anova. Equations and derivation of the Bayesian Anova. [file 1471-2105-12-88-S2.PDF]

Appendix 2: Bayesian Anova

Richard Newton*[1], Jason Hinds[2] and Lorenz Wernisch[1]

[1]MRC Biostatistics Unit, Robinson Way, Cambridge, CB2 0SR, UK
[2]Bacterial Microarray Group, Division of Cellular & Molecular Medicine,St. George's, University of London, Cranmer Terrace, London, SW17 0RE, UK

Email: Richard Newton*- richard.newton@mrc-bsu.cam.ac.uk; Jason Hinds - j.hinds@sgul.ac.uk ; Lorenz Wernisch - lorenz.wernisch@mrc-bsu.cam.ac.uk;

*Corresponding author

We follow the Bayesian treatment of linear models as outlined in Sorensen and Gianola [Sorenson and Gianola(2002)] although the specific forms of posteriors we are interested in deviate slightly from the book.

The objective is to obtain posterior distributions for the coefficients θ in

$$y = X\theta + Zu + \epsilon$$

while integrating out the nuisance variables u and ϵ. As priors we assume $\theta \sim N(0, B\sigma_\theta^2)$, $u \sim N(0, A\sigma_u^2)$, and $\epsilon \sim N(0, I\sigma_e^2)$. It is straightforward to see that, conditioned on θ, the prior predictive distribution of $y - X\theta$ is a Gaussian with mean 0 and covariance matrix $\sigma_e^2\, V = ZAZ'\sigma_u^2 + I_n\sigma_e^2$. That is,

$$p(y \mid \theta, A, \sigma_u^2, \sigma_e^2) \propto (\sigma_e^2)^{-n/2} \exp(-\frac{1}{2\sigma_e^2}((y - X\theta)'V^{-1}(y - X\theta))^{-(\nu+d)/2)})$$
$$V = ZAZ'\frac{\sigma_u^2}{\sigma_e^2} + I_n$$

(we keep track of all variance terms for later use). The posterior distribution for θ is (see equation (6.67) in [Sorenson and Gianola(2002)])

$$p(\theta \mid y, A, \sigma_u^2, B, \sigma_\theta^2, \sigma_e^2) \propto (\sigma_\theta^2)^{-b/2}\,(\sigma_e^2)^{-n/2}\, \exp(-\frac{1}{2\sigma_e^2}((\theta - \hat{\theta})'W^{-1}(\theta - \hat{\theta})))$$
$$W = (X'V^{-1}X + B^{-1}\frac{\sigma_e^2}{\sigma_\theta^2})^{-1}$$
$$\hat{\theta} = WX'V^{-1}y$$

where b is the dimension of θ.

We are left with the problem of integrating out variance components $\sigma_u^2, \sigma_e^2, \sigma_\theta^2$. There is no analytical solution to this integral in its general form. However, making the usual assumption that the error variance σ_e^2 is actually closely

related to the variance factors σ^2_θ and σ^2_u of the coefficients and setting $\sigma^2 = \sigma^2_e = \sigma^2_u = \sigma^2_\theta$, a conjugate analysis is possible for σ^2. We assume a prior $p_{\mathrm{ICh}}(\sigma^2 \mid \nu_0, \sigma^2_0)$. First note that

$$(y - X\theta)'V^{-1}(y - X\theta) + \theta' B^{-1}\theta = (\theta - \hat{\theta})'W^{-1}(\theta - \hat{\theta}) + S_\theta + S_e$$

with

$$S_\theta = \tilde{\theta}' D(D + B^{-1})B^{-1}\tilde{\theta}, \quad S_e = (y - X\tilde{\theta})^2, \quad D = X'V^{-1}X, \quad \tilde{\theta} = D^{-1}X'V^{-1}y$$

where $\tilde{\theta}$ is the ML estimate of θ (after integrating over u). The joint distribution of θ and $\sigma^2 = \sigma^2_e = \sigma^2_u = \sigma^2_\theta$ is

$$p(\theta, \sigma^2 \mid y, A, B) \propto (\sigma^2)^{-(n/2+b/2+\nu_0/2+1)} \exp\left(\frac{(\theta - \hat{\theta})'W^{-1}(\theta - \hat{\theta}) + S_\theta + S_e + \nu_0\sigma^2_0}{2\sigma^2}\right)$$

We obtain

$$p(\theta \mid y, A, B, \nu_0, \sigma^2_0) = \int_0^\infty p(\theta, \sigma_2 \mid y, A, B)\, d(\sigma^2) = p_t(\theta \mid \hat{\theta}, n + \nu_0, (S_\theta + S_e + \nu_0\sigma^2_0)W) \qquad (1)$$

where n is the dimension of y and b is the dimension of θ.

Finally, to derive a likelihood for the optimisation of hyperparameters we start with the predictive likelihood conditioned on the variance components, which is a Gaussian with mean 0 and covariance $\sigma^2_e U = XBX'\sigma^2_\theta + ZAZ'\sigma^2_u + I\sigma^2_e$. For further analysis we assume equality of all variance components and use the same prior as above on σ^2,

$$p(y \mid A, B, \nu_0, \sigma_0) = \int p_{\mathrm{N}}(y \mid A, B, \sigma^2)\, p_{\mathrm{ICh}}(\sigma^2 \mid \nu_0, \sigma^2_0)\, d(\sigma^2) = p_t(y \mid 0, \nu_0, \sigma^2_0(XBX' + ZAZ' + I_n)) \qquad (2)$$

References

Sorenson and Gianola(2002). D Sorenson and D Gianola. *Likelihood, Bayesian, and MCMC Methods in Quantitative Genetics*. Springer, 2002.
